# Evaluation of the Family Health Support Centers focusing on the integration to supported teams

**DOI:** 10.11606/S1518-8787.2018052000122

**Published:** 2018-04-04

**Authors:** Thaís Titon de Souza, Maria Cristina Marino Calvo

**Affiliations:** I Universidade Federal de Santa Catarina. Centro de Ciências da Saúde. Programa de Pós-Graduação em Saúde Coletiva. Florianópolis, SC, Brasil; II Universidade Federal de Santa Catarina. Centro de Ciências da Saúde. Departamento de Saúde Pública. Florianópolis, SC, Brasil

**Keywords:** Health Evaluation, Health Human Resource Evaluation, Patient Care Team, Primary Health Care. manpower, Unified Health System, Avaliação em Saúde, Equipe de Assistência ao Paciente, Avaliação de Recursos Humanos em Saúde, Atenção Primária à Saúde, recursos humanos, Sistema Único de Saúde

## Abstract

**OBJECTIVE:**

To evaluate the Brazilian Family Health Support Centers focusing on the integration to supported teams.

**METHODS:**

This is an evaluation study in which we carried out a documentary analysis and modeling of the intervention focusing on the work integrated to the supported teams, which has allowed us to describe dimensions, objectives, and expected results. We defined the outcome indicators and their respective measures and sources of information. We used consensus techniques with key informants to validate the models and the matrix of indicators.

**RESULTS:**

The evaluation study was appropriate, and it allowed a better definition and knowledge about the intervention.

**CONCLUSIONS:**

There is coherence between the objectives of the Family Health Support Centers and their structure, although there are difficulties to operationalize them. We recommend a formative evaluation of the Family Health Support Centers focusing on the work integrated to the supported teams, seeking to strengthen them to achieve the expected results.

## RESUMO

**OBJETIVO:**

Analisar a avaliabilidade dos Núcleos de Apoio à Saúde da Família com foco na integração às equipes apoiadas.

**MÉTODOS:**

Estudo de avaliabilidade em que foram realizadas análise documental e modelização da intervenção com foco no trabalho integrado às equipes apoiadas, o que permitiu descrever dimensões, objetivos e resultados esperados. Foram definidos indicadores de resultados e suas respectivas medidas e fontes de informação. Foram aplicadas técnicas de consenso com informantes-chave para validação dos modelos e da matriz de indicadores.

**RESULTADOS:**

O estudo de avaliabilidade mostrou-se apropriado, possibilitando melhor definição e conhecimento acerca da intervenção.

**CONCLUSÕES:**

Há coerência entre os objetivos dos Núcleos de Apoio à Saúde da Família e sua estruturação, ainda que existam dificuldades para operacionalizá-los. É recomendado proceder a uma avaliação formativa dos Núcleos de Apoio à Saúde da Família com foco no trabalho integrado às equipes apoiadas buscando fortalecê-los para o alcance dos resultados esperados.

## INTRODUCTION

The Brazilian Family Health Support Centers (NASF) were created in 2008 to expand the scope of the Primary Care (PC), as well as its resolubility^1^. Its composition should be defined based on the health needs of the territory and the supported teams from the Family Health and Primary Care for Specific Populations (FH/PC teams), which share the responsibility for the production of care.

The matrix support guides the integrated work among these teams. Technicalpedagogical and care actions that better respond to the needs of the users or the territory are defined based on agreements between professionals^2^. The performance differs from the outpatient model and the logic of indiscriminate referrals, being guided by the extended care and co-responsibility^3^ ^,^ ^4^.

In order to achieve the expected integration and sharing and consequently the greatest possible degree of quality and resolubility, challenges are identified, such as insufficient mechanisms to monitor and evaluate the results achieved^4^ ^–^ ^6^. Few studies report the results of the actions of the NASF on the supported teams and the population cared for, and they are, in general, related to their implementation or the performance of the different professional categories in PC^7^ ^–^ ^10^. The lack of official data on the work of the NASF hinders the evaluation of the results produced. Its inclusion in the Primary Care e-SUS and in the National Program for Improving Access and Quality of Primary Care (PMAQ) can contribute to overcome this gap.

Therefore, we need mechanisms to evaluate and monitor the NASF that consider the results achieved, especially from the work integrated to the supported teams. This study aimed to evaluate the Family Health Support Centers focusing on the integration to supported teams.

## METHODS

This is an evaluation study (ES), understood as a set of procedures that precede the evaluation stage itself and which point to its usefulness and opportunity, making it more consistent and more credible^11^. Among the expected products, we can mention the complete description of the program or intervention under analysis and the definition of the main issues to be considered in the evaluation^12^.

**Figure 1:**
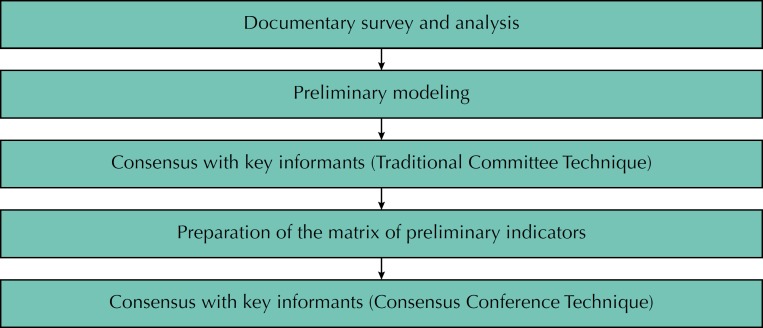
Stages of the design of the evaluation study of the Family Health Support Centers.

We used the qualitative approach. Figure 1 presents the operation flowchart. We carried out a documentary analysis to elaborate the theoretical and logical models of the NASF focused on the work integrated to the supported teams. Based on a review of the documents from the Ministry of Health and specialized literature from the Lilacs (Latin American and Caribbean Literature in Sciences) and SciELO (Scientific Electronic Library Online) databases, we selected nine papers and eighteen scientific papers. The inclusion criteria were: presence of one or more search terms (NASF, Family Health Support Center, matrix support) in the title (equivalent terms are not available in the Health Sciences Descriptors [DeCS]), online availability of the full text, text in Portuguese, and publication period between 2010 and 2015 for articles and between 2004 and 2015 for ministerial documents. We did not consider materials on aspects unique to professional categories that make up the NASF or experiences of matrix support developed by other health teams and the articles that were duplicated in the databases.

After collecting information and clearly defining the object under study, we performed the initial modeling of the intervention. For the consensus of the models, we used the technique of Traditional Committee, with open discussion on a given topic between specialists^13^. We selected twelve key informants, represented by four NASF professionals, two FH/PC team professionals, two health managers, and four health assessment experts, who proposed relevant changes in the presented models. The NASF professionals, for example, reinforced the link of the NASF to the PC and pointed to the need to make clear the expected results of the work integrated to the supported teams.

We prepared a preliminary version of the matrix of indicators validated using the Consensus Conference, a technique that seeks to reconcile the possibility of open discussion and the preservation of the anonymity of the participants^14^. The experts indicated their complete agreement or partial or total disagreement with the elements of the matrix using an electronic questionnaire, suggesting changes. After consolidating the data, we carried out a consensus workshop to discuss issues that did not show agreement in the previous stage^13^. The contributions of the experts, including the NASF professionals, were significant to make the expected results clearer, to redefine indicators initially focused on process evaluation, to qualify measures and questions for the verification of the indicators, and to restructure the matrix to meet the objective of the evaluation of the results achieved by the NASF from the work integrated to the supported teams. Finally, we resent an electronic questionnaire containing the matrix, obtaining a final consensus. Twelve key informants participated in these steps with representation similar to the consensualization phase of the models.

The research was approved by the Research Ethics Committee with Human Beings of the Universidade Federal de Santa Catarina (Opinion 1,248,870/2015), in relation to the ethical precepts. All participants had access and signed the informed consent.

## RESULTS

### Theoretical Model

**Figure 2:**
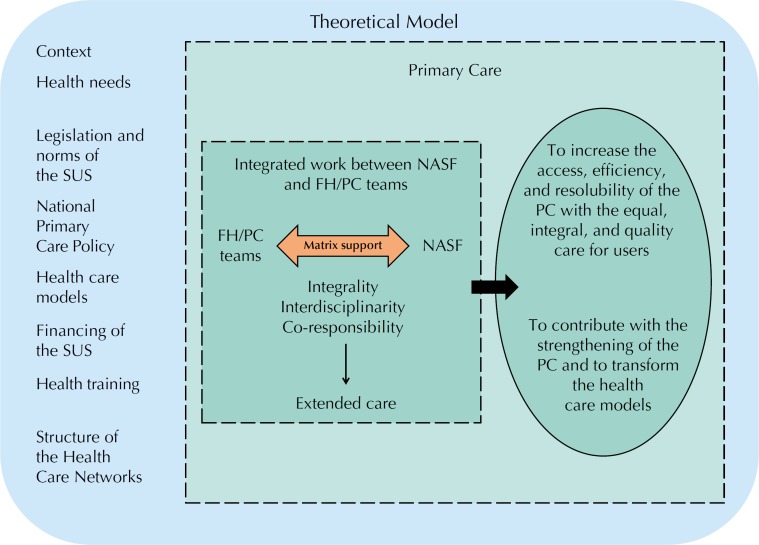
Schematic representation of the theoretical model of the Family Health Support Centers. NASF: Family Health Support Center; FH/PC Teams: teams from the Family Health and Primary Care for Specific Populations; PC: Primary Care; SUS: Brazilian Unified Health System

The theoretical model of the NASF guides how it ideally works to achieve its goals^15^ (Figure 2).

We identified different contextual factors that act directly on PC and interfere in the relation established between the NASF and supported teams. The social and health needs of the population, for example, require managers to identify priorities and define strategies to handle reality. This influences the definition of the constitution of the NASF teams and their way of operating in each territory^2^ ^,^ ^3^ ^,^ ^16^.

The Brazilian Federal Constitution and the legislation governing the SUS, especially the National Primary Care Policy, together with the different existing care models, are also determinants for the relation established. Currently, the biological model predominates. This model guides the work of the FH/PC teams to the individual care of great demand, shortening the time available for discussion, pacing, and joint work. This can pressure practitioners to support its reproduction, leading the NASF to malfunction^17^ ^,^ ^18^. The discrepancy leads to resistance and conflicts and hinders the collaborative work and co-responsibility.

In addition to this, we can also mention the professional training commonly deficient in the matrix support logic, the aspects related to SUS funding that directly interfere with the technical proposal related to the configuration and performance of the NASF (including the number of teams supported and the population cared for), and the structure of the Health Care Networks (HCN), especially of Medium Complexity^4^ ^,^ ^18^ ^,^ ^19^. The incipience of the HCN may distort the proposal of integrated work by leading to the use of the NASF as a replacement of the Networks, directing it only to the specialized work^18^.

Contrary to this logic, the integrated work between teams takes place from the theoretical-methodological framework of the matrix support, considering^2^:

- That the NASF should act in an integrated and collaborative manner with the FH/PC teams at the individual and collective care levels. It must have under its own responsibility the supported team itself, as well as the direct care of the population and the action on the surrounding territory^2^ ^,^ ^3^ ^,^ ^16^;
- Integrity as a precept for the organization of health services;
- Interdisciplinary action, which should promote the disruption of verticalized relations and minimize the occurrence of iatrogenies;
- That the NASF and supported teams should share responsibility for their users and territories, rather than transferring it. This implies co-responsibility, guiding actions developed in contrast to the models based on the fragmented and individualized care^4^; and,
- That the technical-pedagogical and care dimensions of the matrix support are complementary and can be considered as practical aspects of the operationalization of the principles and guidelines of the SUS, especially integrality and interdisciplinarity, resulting in actions based on extended care^16^.

It is hoped that the fragmented and medical logic of the health care can be overcome in favor of the integral care, whose reorientation occurs together with the Family Health Strategy^1^ ^,^ ^20^ ^–^ ^22^. As a result, the NASF should support, improve, and expand the health care, strengthening the attributes of the Primary Health Care (PHC) together with the supported teams^2^ ^–^ ^4^ ^,^ ^7^ ^,^ ^16^ ^,^ ^20^ ^,^ ^23^ ^,^ ^24^.

### Logical Model

It is hoped that the NASF can expand the scope of the PC, increase its ability to respond to health problems and needs, and assist the population demands that are not met by the supported teams^1^ ^,^ ^3^ ^,^ ^4^ ^,^ ^7^ ^,^ ^17^ ^,^ ^20^ ^–^ ^23^. Thus, we decided to elaborate the logical model for the evaluation of its results regarding the broadening of the access and the resolubility of the PC.

**Figure 3:**
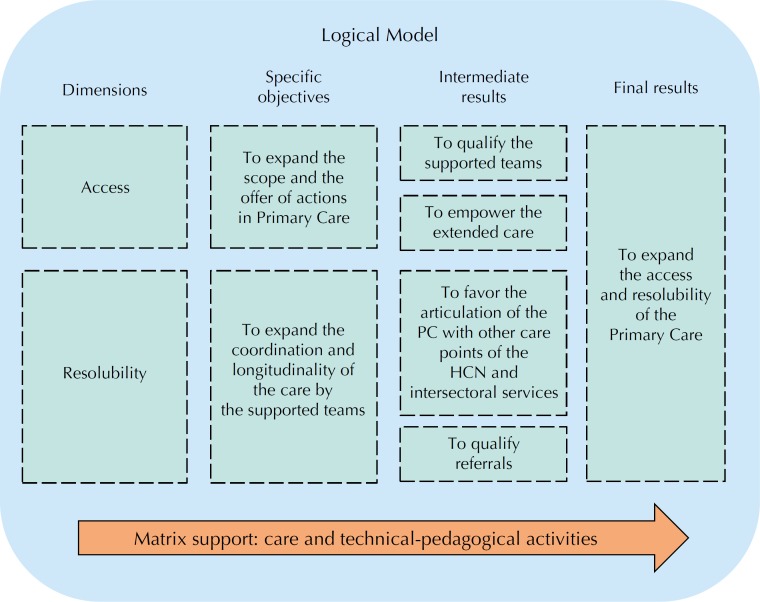
Schematic representation of the logical model of the results achieved by the Family Health Support Centers focused on expanding the access and resolubility in Primary Care. NASF: Family Health Support Center; PC: Primary Care; HCN: Health Care Networks

In order to show how the intervention under study should be implemented, we identified the dimensions, specific objectives, and expected results from the work integrated to the FH/PC teams (Figure 3).

The first dimension of the model presented as intermediary results the qualification of the supported teams and the potential of the extended care^17^ ^,^ ^21^ ^,^ ^22^ ^,^ ^24^ ^,^ ^25^. The technicalpedagogical and care interventions of the matrix support allow the expansion of the scope and offer of actions in PC with the qualification of teams to produce care and to meet previously unmet health needs. The incorporation of the NASF professionals, therefore, in addition to expanding human resources, should enable the offering of actions with greater proximity to the territory and the reality of this population, with the work articulated to the FH/PC teams^1^ ^–^ ^4^ ^,^ ^7^ ^,^ ^16^ ^,^ ^18^ ^,^ ^20^ ^,^ ^23^.

The second dimension, called “Resolubility”, showed the intention that the implementation of the NASF contributes to expand the coordination and longitudinality of the care by the supported teams. To this end, it should favor the articulation of the PC with other points of the HCN and intersectoral services and qualify the referrals^1^ ^,^ ^2^ ^,^ ^25^.

Actions that improve the use of the Secondary and Tertiary Care Network, such as the reorganization of demand and improvement of the relation between the different care points, should promote the rationalization of the access to specialized resources and contribute to expand the ability of the FH/PC teams to coordinate the care of users^2^ ^,^ ^4^ ^,^ ^7^ ^,^ ^17^ ^,^ ^25^. They collaborate to strengthen the attribute of longitudinality, which tends to produce more precise diagnoses and treatments and reduce procedures of greater technological density and unnecessary referrals to experts^26^.

The support of the NASF should therefore favor the continuity and ordering of the care by the PC, strengthening it in the HCN, without disregarding that such results also depend on other factors^2^ ^,^ ^25^. Its contribution to the organization and the reduction of unnecessary referrals to other services is an important action against the limitations of the Network in the various care points^2^ ^,^ ^7^ ^,^ ^18^ ^,^ ^25^.

### Evaluation Matrix

The evaluation matrix of the results of the NASF focused on the work integrated to the supported teams was built based on the theoretical and logical models. It presents the dimensions of “Access” and “Resolubility”, with two sub-dimensions and four indicators each (Box).

**Box:** Evaluation matrix of the results produced by the Family Health Support Centers based on the work integrated to supported teams.

Results produced by the NASF based on the work integrated to supported teams						
Dimension	Intermediate result	Sub-dimension	Indicator	Measures	How to measure	Sources
Access	To qualify the supported teams	Performance of the supported teams	Care practice	• Previous care actions not carried out by the supported teams	Interviews Documentary analysis	FH/PC teams Minutes of meetings, therapeutic projects, and care protocols
				• Clinical conduct of the teams previously supported for the specific care of the NASF		
				• Performance in the territory		
			Health practice	• Performance of the health planning		FH/PC and NASF teams Minutes of the meetings and actions carried out
				• Work focusing on health promotion		
	To empower the extended care	Interdisciplinarity and integrality	Integration and sharing between teams	• Sharing of diagnoses	Interviews	FH/PC and NASF teams
				• Co-responsibility in the definition of actions	Interviews Documentary analysis	FH/PC and NASF teams Schedule of the professionals, joint case management lists, and minutes of meetings
			Actions offered by the NASF	• Actions offered in the Primary Care after implementation of the NASF	Interviews	FH/PC and NASF teams
				• Waiting time for specific care by NASF professionals		
Resolubility	To favor the collaboration of the PC with care points of theHCN and intersectoral services	Coordination of the care	Therapeutic management in Primary Care	• Management of cases shared between NASF and supported teams	Interviews Documentary analysis	FH/PC and NASF teams Schedule of the professionals, management case lists, and minutes of meetings
				• Management of the cases shared in the HCN by the FH/PC teams		FH/PC teams Minutes of meetings, therapeutic projects, and care protocols
			Collaboration of the PC with other point of the HCN and intersectoral service	• Shared care in HCN	Interviews Documentary analysis	FH/PC and NASF teams Minutes of meetings and discussion of cases, therapeutic projects, care flows or protocols, monitoring of referrals, and waiting lists for specialized appointments
				• Care intersectorally shared		FH/PC and NASF teams Minutes of meetings and intersectoral actions, therapeutic projects, and intersectoral care flows or protocols
	To qualify referrals	Longitudinality	Continuity of the clinical relationship in PC	• Health care • Intervention in immediate or unforeseen situations in PC	Interviews	FH/PC and NASF teams
			Clinical resolution	• Referrals to other care points and intersectoral services		FH/PC teams
Total	4	4	8	16	-	-

FH/PC teams: teams from the Family Health and Primary Care for Specific Populations; NASF: Family Health Support Center; PC: Primary Care; HCN: Health Care Networks

In the dimension of “Access”, we considered the double burden of responsibility attributed to the NASF^2^. In the sub-dimension of “Performance of the Supported Teams”, we sought to identify the results of the technical-pedagogical dimension of the matrix support on the ability to produce care by the teams supported by two indicators: Care practice, which seeks to identify the increase of the ability of the teams supported for the effectiveness of the care from the analysis and performance in clinical terms, and Health practice, which aims to identify the increase of the ability of the FH/PC teams for the effectiveness of the care with the analysis and action under the health and collective intervention terms^3^ ^,^ ^7^ ^,^ ^20^ ^,^ ^22^ ^,^ ^25^.

For the sub-dimension of “Interdisciplinarity and Integrality”, we selected the following indicators: Team integration and sharing, which verifies the results achieved on the interdisciplinary, integrated, and collaborative work ability between the supported teams and the NASF for the production of care, and Actions offered by the NASF, which evaluates the results achieved on the expansion of the scope and the access to specific care actions of the NASF in order to respond to the health needs and demands previously unmet by the FH/PC teams.

In the dimension of “Resolubility”, we considered that the actions of the NASF should strengthen the coordination ability and the provision of longitudinal care by the supported teams, as well as the horizontal cooperation and work with other care points. With a logic that privileges the collaborative work, it should increase the potential of the PC as a manager and the main access of the HCN, also increasing its resolubility.

In the sub-dimension of “Coordination of the care”, we expected that the shared responsibility would lead to a review of the referral practice based on the referral and counter-referral, extending it to the sharing of responsibilities^3^. The indicators selected for this sub-dimension (Therapeutic Management in Primary Care and Collaboration of the Primary Care with other points of the HCN and intersectoral services) aimed to identify the results achieved in relation to the shared care management ability of the NASF and the supported teams and other care points, in addition to the ability to develop integrated actions in the Care Networks and intersectoral services.

In the last sub-dimension of “Longitudinality”, we considered that longitudinality requires a regular source of attention and use over time, regardless of the type of problem^26^. The indicator of “Continuity of the clinical relationship in PC” aimed to identify the results of the NASF on the ability of the PC for its accomplishment. This is because it assumes the guarantee of integral care and is one of the aspects that can determine the ability to provide longitudinal care^3^. In the specific context of PC, health care is a strategy to reach personal longitudinality as it is related to the good communication and favors the continuity of the care, so that the NASF can contribute to its consolidation^26^. In addition, it can offer support in unexpected situations or which demand immediate action, considering the ability of each team to handle these situations.

The increase in the number of professionals inserted in the PC would also increase the ability for clinical Resolution, the last indicator presented. In addition to expanding the professional knowledge and practices, the mobility and vision of the NASF regarding the diverse teams favor connections, including the intersectoral ones, and increase therapeutic projects, with potential to improve care networks and flows in the work of these teams^2^. As a consequence, it should contribute to rationalize the access to specialized health services and intersectoral services based on actions shared with FH/PC teams^17^.

Access and resolvability were considered transverse axes of observation of the evaluative matrix insofar as they are presented as expected final results and are simultaneously involved in the two dimensions evaluated. Quality was present in the entire matrix, understood as the ability to meet the attributes of value and merit, that is, to apply properly the resources and to do what is proposed right to meet the needs of the FH/PC teams and users (Scriven, 1991 *apud* Scaratti and Calvo, 2012)^27^. While higher quality is achieved when the final results of the integrated work between the NASF and supported teams are obtained, quality also interferes with the ability of these teams to achieve these results.

## DISCUSSION

The short time of existence of the NASF and the lack of evaluation studies on the subject indicate the importance of research studies that contribute to the understanding of this intervention and to the proposition of questions that direct the formative evaluation of these teams^11^.

In the absence of an explicit and clearly articulated theory, we had to develop the theoretical and logical models of the intervention^11^. The identification of its components was important for the proposition of indicators that seek to favor the progress of the NASF. The selection of key informants with prior knowledge or experience in relation to the subject and health assessment was important to make the intended goal image clearer with the implementation of this team, as well as the way in which the intervention can be implemented to achieve it.

Based on the logical operational analysis, we can see the coherence between objectives and the structure of the NASF, although there are difficulties to operationalize it. We found challenges to achieve the expected integration and sharing from the integrated work between the NASF and the supported teams and, thus, the greater degree of quality and resolubility possible. Some of them are: low understanding about the matrix support and its little practical consolidation, difficulty in defining the work object of the NASF, understanding the NASF as an outpatient specialty service, poor training of workers to act in accordance with the recommended logic, insufficient organizational devices to support the shared work, presence of suppressed demand for some professional categories, and insufficient mechanisms to monitor and evaluate the results achieved^4^ ^–^ ^6^.

The inadequacy of effective registration and monitoring mechanisms that address the actions of the NASF highlights the need to develop measurable and efficient tools for evaluation and management^28^. In order to better understand the work process of the NASF and to define strategies to reach the desired results, appropriate indicators need to be developed. Its insufficiency causes difficulties when guiding the actions towards the integrality and the interdisciplinarity of the care^28^.

The evaluation study (ES) should contribute to improve the development of the intervention analyzed. The theoretical and logical models and indicators proposed in the evaluation matrix are useful tools to support the formulation of policies and the management of the NASF in Brazil. By keeping the relation between them close, they explain the chain of causation that leads to the expected results and suggest mechanisms to identify if they are being reached, indicating their plausibility^29^. The developed ES should therefore contribute to evaluate whether the actions undertaken are moving towards the expected changes with the implementation of these teams in PC^29^.

Mechanisms need to be developed to evaluate and monitor the work of the NASF and the results produced from the integrated work to the supported teams. The FH/PC teams play an important role in consolidating the matrix support. Their organization and understanding of the new relation and care model are paramount to the arrangement of the NASF as a support team in PC and not as an expert service in that care context. The evaluation model presented aims to evaluate the results of the NASF from the work integrated to the supported teams.

The ES showed that the NASF is an evaluable intervention, as we could increase the knowledge about the intervention and define appropriate components and indicators. However, the intervention is not visibly illustrated in the different literatures analyzed, which complement each other but often bring new elements in relation to what is expected from its implementation. There is indicative of a lack of clarity about what is expected to be achieved from its integrated action to the supported teams. This makes the definition of appropriate indicators for the evaluation of these teams mandatory.

The models and matrix presented help advance this issue. The ES is presented as an alternative to qualify the understanding about the NASF and to highlight the identification and discussion of the results achieved, as well as strategies to achieve them.

Other evaluation designs can be developed for the intervention, including focusing on one or another dimension of the matrix support. In this study, we sought to overcome this duality in order to broaden the possibilities of the action of the NASF to reach the quality and resolubility expected from the work integrated to the supported teams.

There is no provision to control the external context of the integrated work between the NASF and supported teams in the proposed matrix, but it should be considered in the evaluation stage. The working conditions of the NASF and supported teams, the structure of the HCN, the composition and implementation time of the NASF, among other contextual factors, should be weighed in the studies that evaluate the results of the NASF from the integration to supported teams in different realities.

The models and the evaluation matrix started from the conception that the relation between these teams is guided by the principles and guidelines of the SUS and PHC, especially by the interdisciplinarity and integrality of the care, promoting co-responsibility and putting into practice the extended care. Considering the recent process of implementation of the NASF and the incipient mechanisms to evaluate the results, especially from the work integrated to the FH/PC teams, the ES can contribute with the qualification of this intervention in Brazil.
